# The Degenerate Ear

**Published:** 1905-10-07

**Authors:** 


### THE DEGENERATE EAR.

It is a habit among medical writers to mention "stigmata of degeneration" as though the expression carried some definite and definable meaning. And yet there probably is not anyone who could give an exact and comprehensive interpretation of the phrase which would bear scientific scrutiny. Most authors who have described particular conformations of the human frame or other manifest features —such, for example, as the texture and colour of the hair, as signifying a tendency towards mental degeneracy in the possessor, appear to have fallen into a variety of errors which to a large extent invalidate their conclusions. Several of these errors are demonstrated by Dr. V. V. Vorobieff,[1] of Moscow, who has made a somewhat extensive inquiry into the conformation of ears both in insane and normal members of the community. He mentions the following as common fallacies: Lack of comparison between pathological and normal groups of people, and the unsubstantiated assertion that certain given peculiarities do not exist in normal subjects; neglect of racial peculiarities, of social and economic differences, and of the age-factor. We have not space to enter into a detailed account showing the degree to which these errors have been operative, and anyone especially interested in the subject should refer to Dr. Vorobieff's original paper. But we may point out that the results of his inquiry seem to indicate that the commonly accepted stigmata of degeneration, so far as the ear is concerned, do not have the morbid significance which has been attached to them.

The chief aural characteristics which have been regarded as suggestive of mental degeneracy fall into three groups—namely, adherent lobule, Darwin's ear, and undeveloped helix. Dr. Vorobieff finds that in normal individuals these anomalies are quite as prevalent as they are among the insane and criminals.

The frequency of adherent lobule varies considerably in different races of man. Thus it is commoner in Russia than in Western Europe, and is commoner in the inhabitants of Western Europe than among negroes. It cannot, therefore, be regarded as an index to the mental status of either the individual or the race.

But there are two types of ear which do appear to Dr. Vorobieff to be more common among criminals and insane people than among normal individuals of the same race. The first of these is the ear which shows congenital deficiencies or malformations, such as fissures and colobomata of the lobule and other parts, and excrescences often representing rudimentary additional outer ears. Such conditions he thinks entitled to the designation of stigmata of degeneration, for they are much the most commonly met with in association with psychical anomalies.

The second type of ear which was found with especial frequency among mental degenerates was the "standing out" ear, but in this case the condition is perhaps due to rickets, and its occurrence among the insane and the wicked is very likely to be explained by the frequency with which rickets occurs among those classes which have the privilege of producing the great majority of lunatics and criminals. The question is an interesting one, and might well repay further research.

[1] Jour. Ment. Path., No. 2, 1905.

